# Conserved but not critical: Trafficking and function of Na_V_1.7 are independent of highly conserved polybasic motifs

**DOI:** 10.3389/fnmol.2023.1161028

**Published:** 2023-03-16

**Authors:** Sidharth Tyagi, Nivedita Sarveswaran, Grant P. Higerd-Rusli, Shujun Liu, Fadia B. Dib-Hajj, Stephen G. Waxman, Sulayman D. Dib-Hajj

**Affiliations:** ^1^Medical Scientist Training Program, Yale School of Medicine, New Haven, CT, United States; ^2^Center for Neuroscience and Regeneration Research, West Haven, CT, United States; ^3^Department of Neurology, Yale School of Medicine, New Haven, CT, United States; ^4^Center for Restoration of Nervous System Function, VA Connecticut Healthcare System, West Haven, CT, United States

**Keywords:** sodium channels, channel trafficking, polybasic motifs, electrophysiology, pain, Rab GTPases

## Abstract

Non-addictive treatment of chronic pain represents a major unmet clinical need. Peripheral voltage-gated sodium (Na_V_) channels are an attractive target for pain therapy because they initiate and propagate action potentials in primary afferents that detect and transduce noxious stimuli. Na_V_1.7 sets the gain on peripheral pain-signaling neurons and is the best validated peripheral ion channel involved in human pain, and previous work has shown that it is transported in vesicles in sensory axons which also carry Rab6a, a small GTPase known to be involved in vesicular packaging and axonal transport. Understanding the mechanism of the association between Rab6a and Na_V_1.7 could inform therapeutic modalities to decrease trafficking of Na_V_1.7 to the distal axonal membrane. Polybasic motifs (PBM) have been shown to regulate Rab-protein interactions in a variety of contexts. In this study, we explored whether two PBMs in the cytoplasmic loop that joins domains I and II of human Na_V_1.7 were responsible for association with Rab6a and regulate axonal trafficking of the channel. Using site-directed mutagenesis we generated Na_V_1.7 constructs with alanine substitutions in the two PBMs. Voltage-clamp recordings showed that the constructs retain wild-type like gating properties. Optical Pulse-chase Axonal Long-distance (OPAL) imaging in live sensory axons shows that mutations of these PBMs do not affect co-trafficking of Rab6a and Na_V_1.7, or the accumulation of the channel at the distal axonal surface. Thus, these polybasic motifs are not required for interaction of Na_V_1.7 with the Rab6a GTPase, or for trafficking of the channel to the plasma membrane.

## 1. Introduction

Chronic pain is a massive global health burden, with estimated healthcare costs related to pain ranging from 261 to 300 billion USD in the United States alone (Gaskin and Richard, 2012). In spite of this, currently available therapeutic options for chronic pain are limited and have a spectrum of adverse effects (Hylands-White et al., 2017; Somogyi et al., 2022). Alternative treatments for pain that are specific, non-addictive, and effective are an urgent need.

Underlying pain sensation in most human pain conditions is sodium flux through voltage-gated sodium channels in peripheral sensory neurons and subsequent action potential propagation. These action potentials spread along the length of the axons of primary afferents that form the first synapse with neurons in the dorsal horn of the spinal cord (Basbaum et al., 2009; Dib-Hajj et al., 2010; Bennett et al., 2019). Of the voltage-gated sodium channel family, Na_V_1.7–1.9 are the principal channel isoforms found in unmyelinated peripheral sensory neurons (Dib-Hajj et al., 2010; Bennett et al., 2019; Goodwin and McMahon, 2021), most of which are nociceptors. Na_V_1.7 contributes 60–70% of the TTX-S sodium current in small DRG neurons and acts as a threshold channel that sets the gain on nociceptive firing (Dib-Hajj et al., 2005; Shields et al., 2012; Vasylyev et al., 2014; Chen et al., 2018).

Na_V_1.7 is an attractive therapeutic target for pain because of its key role in regulating electrogenesis and its robust genetic validation in human pain disorders (Bennett et al., 2019; Dib-Hajj and Waxman, 2019). Despite this compelling evidence, existing compounds (principally small molecules) that aim to directly inhibit sodium conductance through the channel have shown a combination of off-target effects and limited efficacy (Alsaloum et al., 2020). An alternative strategy might be to interrupt the trafficking of the channels to the plasma membrane to reduce the excitability of nociceptive neurons and ameliorate pain symptoms. Although Na_V_1.7 has been found all along the primary afferents from the peripheral terminals in the epidermis to the central terminals in the spinal cord (Black et al., 2012), studies of fixed tissues are static; thus new approaches are needed to investigate dynamic regulation of channel transport to axonal ends where action potential initiation and synaptic transmission of nerve impulse occur.

We have recently developed novel full-length Na_V_ constructs that carry a fluorescent tag and used them in a powerful imaging assay, OPAL (optical pulse-chase axonal long imaging), to study the dynamics of Na_V_ transport in real time (Akin et al., 2019, 2021; Higerd-Rusli et al., 2022, 2023). This assay has shown that Na_V_1.7 channels, anterograde trafficking, and density at the cell-surface are upregulated under conditions that mimic aspects of inflammation and in chemotherapy induced peripheral neuropathy (Akin et al., 2019, 2021). Notably, these studies have demonstrated that Na_V_1.7 co-localizes in vesicles with proteins involved in vesicular transport – specific isoforms of Rab GTPases (Akin et al., 2019). However, the molecular determinants of the specificity of this association are not known.

Rab GTPases are molecular switches of the Ras superfamily of small GTPases (Hutagalung and Novick, 2011; Zhen and Stenmark, 2015; Farinha and Matos, 2017; Homma et al., 2021). They alternate between a GTP-bound active state and a GDP-bound inactive form (Seabra and Wasmeier, 2004; Pfeffer, 2013). Rab proteins are central regulators of vesicular trafficking–with different isoforms being critical in all parts of the trafficking pathway (vesicle loading, endosome formation, recycling, insertion, etc; Jordens et al., 2005; Corbeel and Freson, 2008; Liu and Storrie, 2012; Bucci et al., 2014; Wandinger-Ness and Zerial, 2014). There are 60 members of the Rab family of proteins in humans, and 47 are expressed in peripheral DRG neurons (Usoskin et al., 2015). We evaluated the co-trafficking of the most abundantly expressed Rab proteins and have shown that Na_V_1.7 preferentially co-localizes with the Rab6a isoform in anterograde vesicles (Akin et al., 2019). Previous studies (Parmar and Duncan, 2016) investigated the interaction between the viral transmembrane protein p14 and its predominant Rab partner (Rab11a). Rab11a is required for anterograde delivery of this transmembrane protein to the plasma membrane. The authors identified a 7-residue polybasic motif that is composed of two tribasic clusters separated by an acidic residue. Substitution of this polybasic motif with alanine residues resulted in a near-complete absence of the protein at the plasma membrane surface and retention in the Golgi apparatus. Addition of basic charges to the mutants at sites separate from the original motifs rescued plasma membrane expression. Similar patterns are found in mammalian proteins as well. For example, in the β2 adrenergic receptor, basic residues in the C-terminal tail are critical for the interaction with the Rab protein partner (Parent et al., 2009). These findings support the view that polybasic motifs in transmembrane proteins can act as sequence-independent electrostatic interaction motifs that facilitate vesicular transport by Rab GTPases (Parmar et al., 2014a,b).

In this study, we sought to determine whether two polybasic motifs in the cytoplasmic loop that joins domains I and II (L1) of Nav1.7 play a role in the trafficking of Na_V_1.7 in Rab6a positive vesicles. We show that channel constructs with alanine substitutions of basic residues in these motifs do not demonstrate altered co-trafficking with Rab6, vesicular dynamics, or surface expression.

## 2. Materials and methods

Animal studies followed a protocol approved by the Institutional Animal Care and Use Committee at the Department of Veterans Affairs West Haven Medical Center. DRG neurons isolated from 0 to 5-day-old Sprague–Dawley rat pups were transfected with Halo-Na_V_1.7 and EGFP-Rab6a as described previously (Akin et al., 2019; Higerd-Rusli et al., 2022). DRG neurons were isolated from 2–4 day old Sprague Dawley rat pups as previously described in Supplemental material (Dib-Hajj et al., 2009).

### 2.1. Molecular biology

All human Na_V_1.7 plasmids were rendered TTX-resistant (hNa_V_1.7-R) by substituting amino acid (a.a.) Tyr362 with serine (Y362S) using QuikChange Lightning site-directed mutagenesis (Yang et al., 2016).

**Figure 1:**
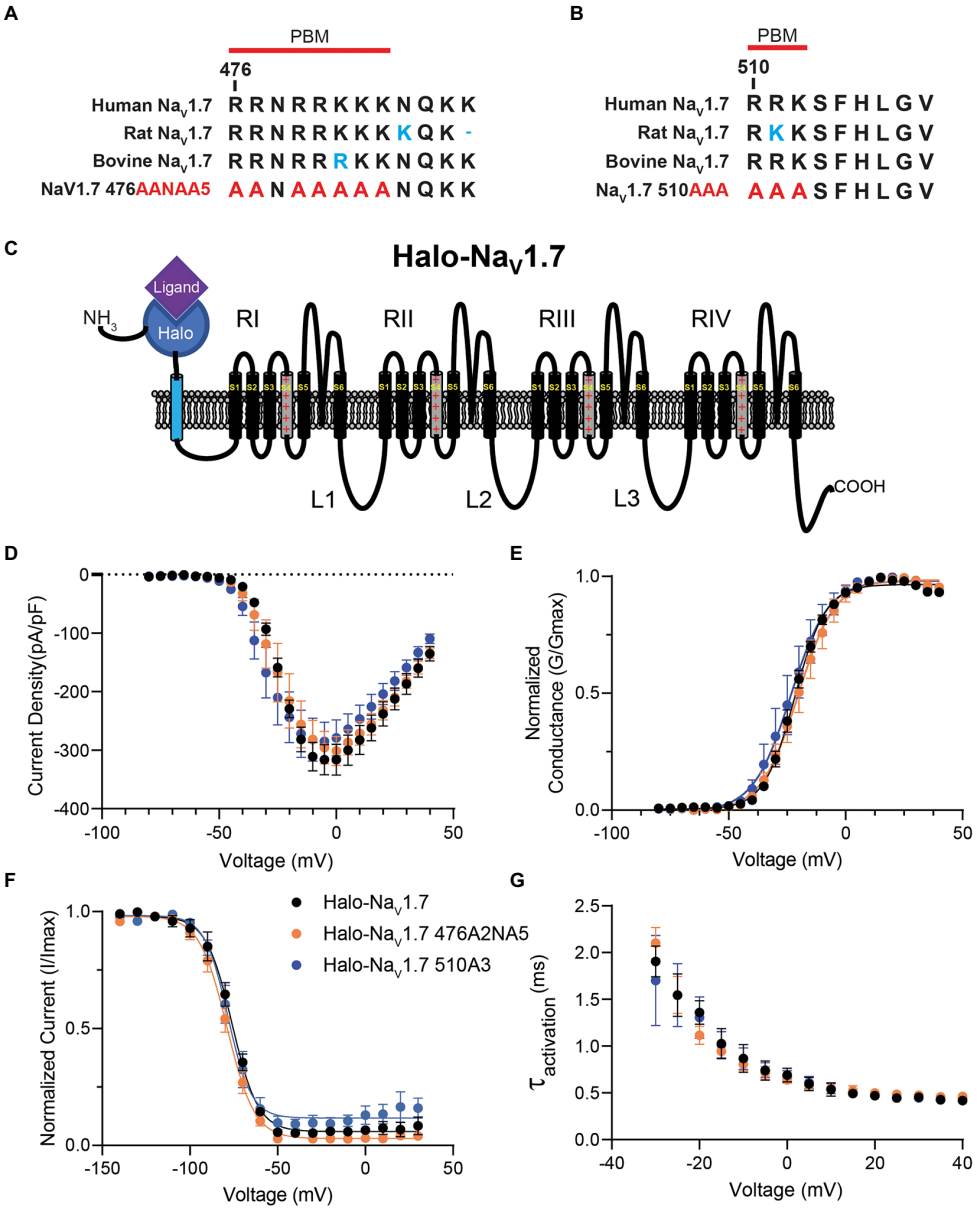
Sequence comparison and electrophysiologic characterization of the polybasic amino acid motifs in Na_V_1.7 loop 1. **(A)** Sequence alignment of Amino Acids 476–487 in loop 1 of Na_V_1.7 between human Na_V_1.7 (accession no. Q15858), rat Na_V_1.7 (accession no. O08562), bovine Na_V_1.7 (accession no. M0QVY7) and the mutant human Na_V_1.7 with alanine substitutions for basic residues at positions 476–477 and 479–483. Red line marks the definition of the polybasic motif beginning at position 476. **(B)** Sequence alignment of Amino Acids 510–518 in loop 1 of Na_V_1.7 between human Na_V_1.7 (accession no. Q15858), rat Na_V_1.7 (accession no. O08562), bovine Na_V_1.7 (accession no. M0QVY7) and the mutant human Na_V_1.7 with alanine substitutions for basic residues at positions 510–512. Red line marks the definition of the polybasic motif beginning at position 510. **(C)** Schematic of the Halo-Na_V_1.7 construct used in our experiments. The channel features the Halo-tag enzyme fused to the extracellular N-terminus of the channel *via* an extra transmembrane linker. **(D)** HEK293 cells were co-transfected with either Halo-Na_V_1.7, Halo-Na_V_1.7 476A2NA5, or Halo-Na_V_1.7 510A3 and GFP. Positively transfected cells were identified by fluorescence and analyzed by whole-cell voltage-clamp. Current–voltage relationships for currents produced by Halo-Na_V_1.7 (*n* = 10), Halo-Na_V_1.7 476A2NA5 (*n* = 10), or Halo-Na_V_1.7 510A3 (*n* = 10). **(E)** Conductance values were generated by equation 2, normalized to the maximum conductance and fit with equation 3 (Supplementary Material). Half-activation values were similar between the three constructs (*p* = 0.41). **(F)** Fast inactivation relationships for Halo-Na_V_1.7 (*n* = 8), Halo-Na_V_1.7 476A2NA5 (*n* = 7), or Halo-Na_V_1.7 510A3 (*n* = 7) were fit with by equation 3 (Supplementary Material). Half-inactivation values were similar between the three constructs (*p* = 0.40). **(G)** Activation kinetics for the three constructs. The activation phase was fit with Equation 4 (Supplementary Material). Activation time constants were similar for the three constructs (*p* = 0.68). Error bars indicate SEM.

We generated a codon-optimized Halo-Na_V_1.7 construct as described previously (Figure 1C; Akin et al., 2019; Higerd-Rusli et al., 2022). The final construct topology is in order from the N-terminus: 1–30 a.a. β4 signal peptide, 3× myc tag (EQKLISEEDL), Halo-tag enzyme (297 a.a.; Promega), 3× HA tag (YPYDVPDYA), 21 a.a. transmembrane segment (β4 163–183), 7 a.a. linker (SGLRSAT), hNa_V_1.7-R. This channel retains WT gating properties and enables similar current density (Higerd-Rusli et al., 2022).

Alanine substitutions of the polybasic motifs RRNRRKKK at a.a. position 476 and RRK at a.a. position 510 were carried out using QuikChange Lightning site-directed mutagenesis. The mutation of the poly basic motifs yielded the following constructs: Halo-Na_V_1.7 476A2NA5_510A3, Halo-Na_V_1.7 476A2NA5, and Halo-Na_V_1.7 510A3. Identity of the inserts was confirmed by Sanger sequencing.

EGFP-Rab6A (Addgene plasmid # 49469), was obtained from Addgene as a gift from Marci Scidmore (Choudhury et al., 2002).

### 2.2. Optical pulse-chase axonal long-distance imaging

The optical pulse-chase axonal long-distance (OPAL) imaging (Akin et al., 2019) technique was used to selectively label anterograde vesicles containing Halo-Na_V_1.7 during long-distance axonal trafficking as described in detail below:

Images were acquired using an Andor Dragonfly spinning disk confocal microscope built on a Nikon Eclipse Ti fluorescence microscope. Images were collected using an Andor iXon Ultra 888 EMCCD camera through a Lambda 60x oil objective. The system includes an Andor Integrated Laser Engine containing 100 mW 405 nm, 150 mW 488 nm, 150 mW 561 nm, and 140 mW 637 nm solid state lasers. The Nikon Perfect-Focus system was used to maintain focus in the z-plane during timelapse imaging.

All Halo-tag ligand conjugated Janelia Fluor labels (Grimm et al., 2015; Jonker et al., 2020) were generous gifts of L.D. Lavis and J.B. Grimm (Janelia research campus). To label cytoplasmic, somatic Halo-Na_V_1.7, the culture media in the somatic compartment of the microfluidic chamber was removed by washing with DRG-Neuronal Imaging Saline (NIS). DRG-NIS contained (in mM): 136 NaCl, 3 KCl, 1 MgSO_4_, 2.5 CaCl_2_, 0.15 NaH_2_PO_4_, 0.1 Ascorbic Acid, 20 HEPES, 8 Dextrose (pH 7.4 with NaOH, adjusted to 320 mOsm/l). Cell permeable HaloTag-JF646 ligand (100 nM) was added to the somatic chamber for 15 min, and then removed by washing the chamber 3x with NIS. The axonal chamber was also washed with NIS 3x to remove culture media.

Positively transfected axons were identified by excitation with the 488 nm (for eGFP-Rab6a) and the 637 nm (for Halo-Na_V_1.7) channels of the Andor laser system. Time-lapse movies were acquired in the distal axon chamber for up to 1 h after imaging. Green fluorescence from eGFP-Rab6a in the field of view (FOV) was photobleached using the 488 nm laser before movie acquisition. Live image sequences were obtained on the Dragonfly spinning disk by sequentially acquiring images alternating between the green (488 nm) and far red (637 nm) channels. Exposures were 100-ms.

Axons were used for analysis only if the Rab signal showed discrete vesicular movement, i.e., kymographs demonstrating anterograde motion over time. It was then confirmed that the axon contained at least one clearly visible moving Halo-Na_V_1.7 vesicle. Vesicle movement was analyzed using the KymographClear and KymographDirect packages in ImageJ (Mangeol et al., 2016). Time-lapse movies were loaded into ImageJ, and the KymographClear plugin was used to create kymographs from sections of axons. Axons chosen for analysis were isolated from other axons and had multiple clearly visible vesicles that move through the region during the image sequence. Seven axons were analyzed from each of three independent cultures of each condition.

A macro written in the ImageJ scripting language was used to rapidly generate kymographs in a semi-automated fashion. The code is included in the Supplemental material and online in our public GitHub repository.^1^

To analyze vesicular flux and velocity, the kymographs were analyzed using the automated kymograph analysis software KymoButler (Jakobs et al., 2019). Vesicle tracks were automatically detected, and flux was defined by the number of forward moving vesicle tracks that passed the center of the kymograph. Velocity of each vesicle track was measured in μm/s. Data was imported into Python for statistical analysis.

### 2.3. Cell surface labeling

To label Halo-Na_V_1.7 channels at the cell surface, the culture media in the axonal compartment of the microfluidic chamber was removed by washing with DRG-NIS. Cell-impermeable HaloTag-JF635i Ligand (100 nM) was added for 12 min. Axons were washed three times with DRG-NIS, then taken for confocal fluorescence imaging with the imaging system described above.

### 2.4. Voltage clamp recordings

Validation of biophysical properties of the tagged channel was performed by manual patch-clamp in HEK293 cells. HEK293 cells were transiently transfected with plasmids encoding Halo-Na_V_1.7, Halo-Na_V_1.7 476A2NA5, and Halo-Na_V_1.7 510A3 using the LipoJet *In Vitro* Transfection Kit. Cells were simultaneously co-transfected with plasmids encoding eGFP. The day after transfection, cells were trypsinized and replated onto 6 mm coverslips. Successfully transfected cells were identified by robust green fluorescence and used in experiments ~24 h later. The gating properties of Halo-Na_V_1.7 476A2NA5_510A3 channels were evaluated by automated voltage-clamp of transiently transfected HEK cells. Plasmids encoding Halo-Na_V_1.7 476A2NA5_510A3 or WT Halo-Na_V_1.7 (as the control condition) were introduced into HEK 293 cells *via* lipid-based transfection (Lipofectamine 3,000, Thermo Fisher Scientific). Cells were harvested into a cell suspension 48 h post-transfection for recordings by an automated electrophysiology robot (Qube 384, Sophion Biosciences). A step-by-step protocol for this type of high-throughput protocol is described elsewhere (Ghovanloo et al., 2018; Hasan et al., 2021; Ghovanloo et al., 2022).

All electrophysiological experiments were performed at room temperature. Detailed description of electrophysiological protocols can be found in the Supplemental material.

### 2.5. Statistics and figures

Statistical calculations were performed in Python 3.9 equipped with the Pandas package. For any dataset undergoing group comparison testing, the underlying distribution was tested for normality *via* the Kolmogorov–Smirnov test. Non-normally distributed data were evaluated by non-parametric statistical tests, while normally distributed data were evaluated by parametric statistical methods (the specific tests used are indicated in the text). The α-level was set at 0.05.

Figures were generated in GraphPad Prism 9.4.1 (Graphs; GraphPad Software), Fiji (Kymographs, Images of axons (Schindelin et al., 2012)), Adobe Illustrator (Schematics; Adobe Inc), and BioRender (Schematics; www.biorender.com), then assembled in Adobe Illustrator.

## 3. Results

### 3.1. Polybasic motif mutants in L1 do not alter gating properties of human Na_V_1.7 channels

The L1 cytoplasmic loop of Nav1.7 channels contains the sequence RRNRRKKK at position 476 and the sequence RRK at position 510 (Figures 1A,B). These polybasic motifs were mutated by substituting the positively charged residues with alanine. To see if this mutagenesis resulted in changes in channel gating or biophysics, HEK293 cells were transiently transfected with plasmids encoding Halo-Na_V_1.7, Halo-Na_V_1.7 476AANAAAAA (476A2NA5), or Halo-Na_V_1.7 510AAA (510A3; Figures 1A,B). Cells were simultaneously co-transfected with plasmids encoding eGFP. Successfully transfected cells were identified by green fluorescence 24 h post-transfection.

The whole-cell voltage clamp technique was employed to see how mutation of polybasic motif residues at positions 476 and 510 of Na_V_1.7 affects channel gating. There were no significant differences in peak current density (*p* = 0.74 by One-way ANOVA; Figure 1D) between the three clones (I_peak_: −316.3 ± 26.4 (Halo-Na_V_1.7, *n* = 10), −301.2 ± 24.7 (Halo-Na_V_1.7 476A2NA5, *n* = 10), −285.1 ± 32.7 pA/pF (Halo-Na_V_1.7 510A3, *n* = 10). Representative current traces are available in the Supplementary Figure S1A.

Normalized conductance values were fit with the Boltzmann equation (equation 2; Supplementary material) to characterize the voltage-dependence of activation. The current–voltage relationship of all three clones was similar relative to WT (V_1/2_ activation: −21.7 ± 0.6 (Halo-Na_V_1.7, *n* = 10), −20.4 ± 0.6 (Halo-Na_V_1.7 476A2NA5, *n* = 10; *p* = 0.41), and − 23.52 ± 0.9 mV (Halo-Na_V_1.7 510A3, *n* = 10; *p* = 0.19); *p*-values by one-way ANOVA with Tukey’s post-hoc; Figure 1E).

The voltage-dependence of fast-inactivation was assessed by applying 500 ms inactivating pulses from a range of voltages followed by a step to the peak of activation. Normalized conductance values were fit with the Boltzmann equation (equation 2; Supplementary material) and showed similar half-maximal inactivation values between the three clones (V_1/2inact_: −76.0 ± 1.4, −78.9 ± 1.2, −78.3 ± 2.1 for Halo-Na_V_1.7 (*n* = 8), Halo-Na_V_1.7 476A2NA5 (*n* = 7), or Halo-Na_V_1.7 510A3 (*n* = 7) respectively; *p* = 0.40 by one-way ANOVA; Figure 1F).

The activation phases of current traces were fit with a single exponential function (equation 3; Supplementary material) to determine the kinetics of activation. Time constants of activation were not statistically different between the three clones (t_act:_ 16.3 ± 1.7 (Halo-Na_V_1.7, *n* = 10); 14.7 ± 1.2 (Halo-Na_V_1.7 476A2NA5, *n* = 10); 16.5 ± 1.8 (Halo-Na_V_1.7 510A3, *n* = 10); *p* = 0.68 by one-way ANOVA; Figure 1G).

### 3.2. Mutation of polybasic motifs 476RRNRRKKK and/or 510RRK does not abrogate co-trafficking with Rab6A

We have developed OPAL (Optical pulse-chase axonal long-distance) imaging to visualize the movement of individual vesicles carrying proteins of interest down the length of an axon (Akin et al., 2019, 2021; Higerd-Rusli et al., 2022). Halo-Na_V_1.7 covalently binds fluorescent synthetic ligands to allow optical detection of the protein (Los et al., 2008).

**Figure 2:**
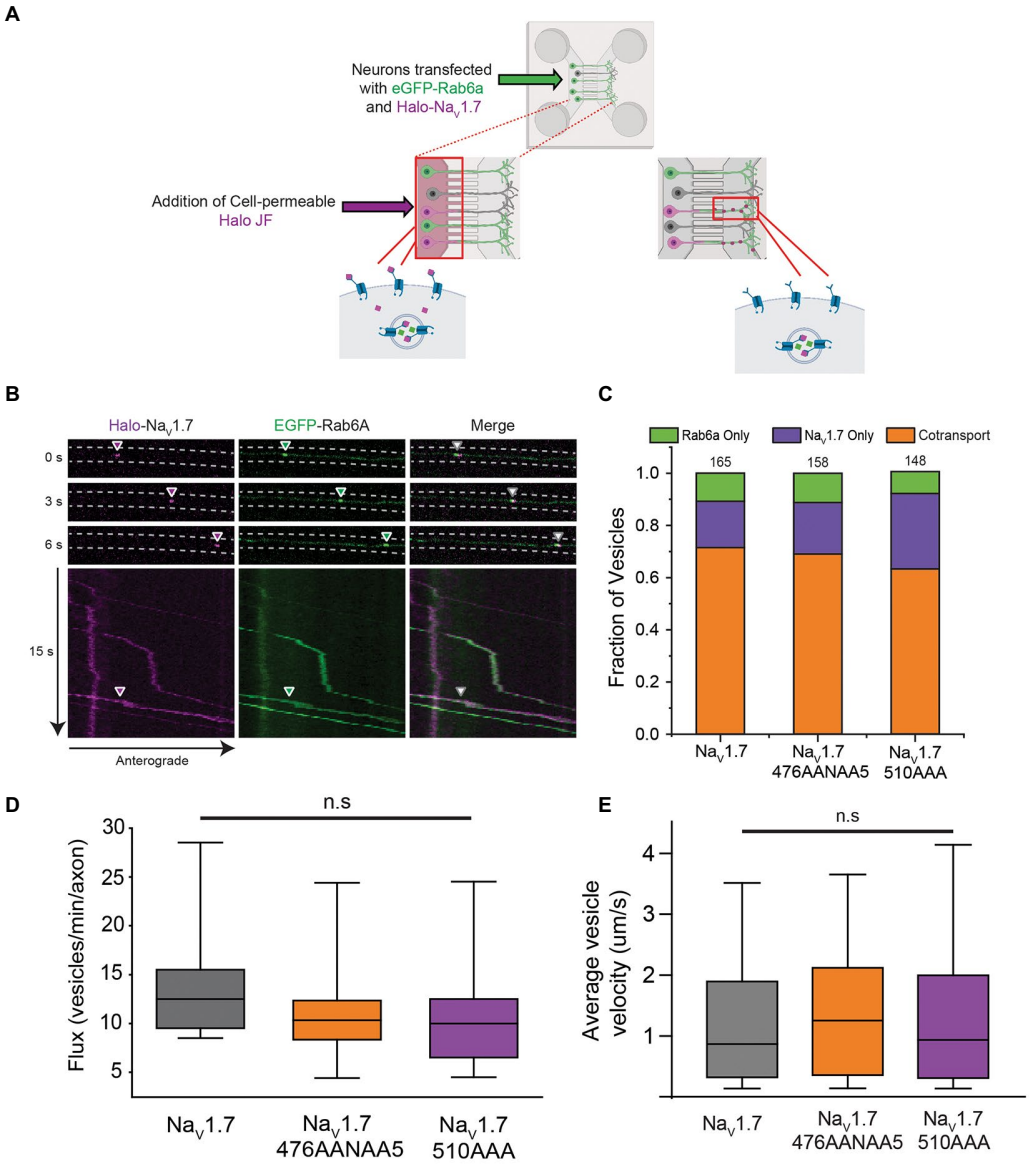
Co-transport of Na_V_1.7 polybasic motif mutants with Rab6a. OPAL imaging was used to visualize anterograde vesicles carrying Halo-Na_V_1.7 constructs labeled with JF646-Halo. Two-color time lapse imaging was performed to reveal vesicles carrying one or both of Halo-Na_V_1.7 or EGFP-Rab6a **(A)** Schematic representation of the OPAL technique used to visualize channel trafficking. **(B)** Selected frames from a time lapse movie generated by a spinning disk confocal microscope where cells were excited by the 488 nm and 637 nm channels of the laser system. Arrowheads show the position of an anterograde vesicle containing Halo-Na_V_1.7 (purple) and EGFP-Rab6a (green). Merged images show overlap of the green and purple signals indicating co-movement. The corresponding kymographs show the vesicle track, and the merged kymograph shows identical vesicle movement for both channels. **(C)** The fraction of vesicles that demonstrate cotransport of Na_V_1.7 and Rab6a are not affected by mutation of either polybasic motif. Two-color time lapse imaging was performed. Kymographs were created for each construct-Rab6a pair, and vesicle tracks were visually inspected and classified as Rab only, Na_V_1.7 only, or both (*p* = 0.51; *n* = 148–165 vesicles per channel, 3 cultures, 7 axons per culture). **(D)** The flux of vesicles in neurons transfected with the polybasic motif mutant constructs was not different than in neurons transfected with WT Halo-Na_V_1.7 (*p* = 0.31; *n* = 300–400 vesicles per channel, 3 cultures, 7 axons per culture). Vesicle tracks were automatically detected by the KymoButler software. Vesicles were counted in the flux measurement if they passed through the midpoint of the kymograph. Box plot bars indicate 5th percentile, 25th percentile, median, 75th percentile, and 95th percentile, in ascending order. **(E)** The velocity of vesicles in neurons transfected with the polybasic motif mutant constructs was not different than in neurons transfected with WT Halo-Na_V_1.7 (*p* = 0.52; *n* = 300–400 vesicles per channel, 3 cultures, 7 axons per culture). Velocity was automatically calculated by the KymoButler software, and reported in mm/s. Box plot bars indicate 5th percentile, 25th percentile, median, 75th percentile, and 95th percentile, in ascending order. ”ns” stands for non-significant (*p*>0.05).

To evaluate whether the L1 polybasic motifs are important for co-trafficking of Na_V_1.7 with Rab6A, we co-transfected neurons with eGFP-Rab6a and either Halo-Na_V_1.7, Halo-Na_V_1.7 476A2NA5, or Halo-Na_V_1.7 510A3 (Figure 1). After 6 days in culture, we visualized anterogradely moving vesicles containing the channel constructs by labeling somatic channels with the cell permeable JF646-HaloTag Ligand and imaging the channels as they are trafficked into the axonal compartment (Figure 2A). Rapid laser and color filter switching enabled the detection of vesicles containing these two protein constructs simultaneously. Only axons expressing both labeled proteins were analyzed for the co-trafficking experiments. Time-lapse imaging of these axons reveals a population of vesicles containing both Halo-Na_V_1.7 and eGFP-Rab6a that can be identified by fluorescence generated by both a green (488 nm excitation) and far-red (637 nm excitation) laser (Figure 2B). The example vesicle in Figure 2B shows an anterogradely moving vesicle expressing both WT Halo-Na_V_1.7 (Figure 2B, left panels) and eGFP-Rab6a (Figure 2B, middle panels). Merged images show significant overlap between fluorescence in both far-red and green wavelengths (Figure 2B, right panels). Kymographs generated from these time lapse movies allow for categorization of any given vesicle as Halo-Na_V_1.7 containing, eGFP-Rab6a containing, or containing both Halo-Na_V_1.7 and eGFP-Rab6a (Figure 2B, bottom panels). The lack of complete overlap between the green and far-red images can be explained by the fact that the green channel is imaged first and there is a 333 ms delay between acquisition of the green image and the far-red. The identical trajectories, fixed delay, and close localization of the two fluorescent signals strongly suggest that the two proteins are trafficked together in the same vesicle.

For DRG neurons expressing the WT Halo-Na_V_1.7 channel, our results were similar to those previously reported, with 71% of anterogradely-traveling vesicles being doubly positive for both Halo-Na_V_1.7 and eGFP-Rab6a. 18% of vesicles were positive for only Halo-Na_V_1.7, while 11% of vesicles were positive for only eGFP-Rab6a (Figure 2C; *n* = 165 vesicles, 21 axons, 3 cultures). DRG neurons expressing the Halo-Na_V_1.7 476A2NA5 channel demonstrated similar vesicle composition, with 69% of vesicles doubly positive for Halo-Na_V_1.7 476A2NA5 and eGFP-Rab6a, 20% positive for only Halo-Na_V_1.7 476A2NA5, and 11% of vesicles positive only for eGFP-Rab6a (Figure 2C; *n* = 158 vesicles, 21 axons, 3 cultures). In DRG neurons expressing the Halo-Na_V_1.7 510A3 channel, 63% of vesicles were doubly positive for Halo-Na_V_1.7 510A3 and eGFP-Rab6a, 28% positive for Halo-Na_V_1.7 alone, and 9% positive for eGFP-Rab6a alone (Figure 2C; *n* = 148 vesicles, 21 axons, 3 cultures). There was no statistically significant difference in vesicle composition between DRG neurons expressing the three channel constructs (*p* = 0.51 by χ^2^ test).

Previously, Parmar et al. (2014b) and Parmar and Duncan (2016) showed that ablation of only one of two tribasic motifs in a protein of interest (p14) did not abolish trafficking of the protein to the plasma membrane or co-trafficking with its complementary Rab protein. If these polybasic motifs are sequence independent, electrostatic interaction motifs, then perhaps the remaining unmutated polybasic motif (476RRNRRKKK in the 510A3 mutant or 510RRK in the 476A2NA5 mutant) provided enough charge to facilitate Golgi export.

**Figure 3:**
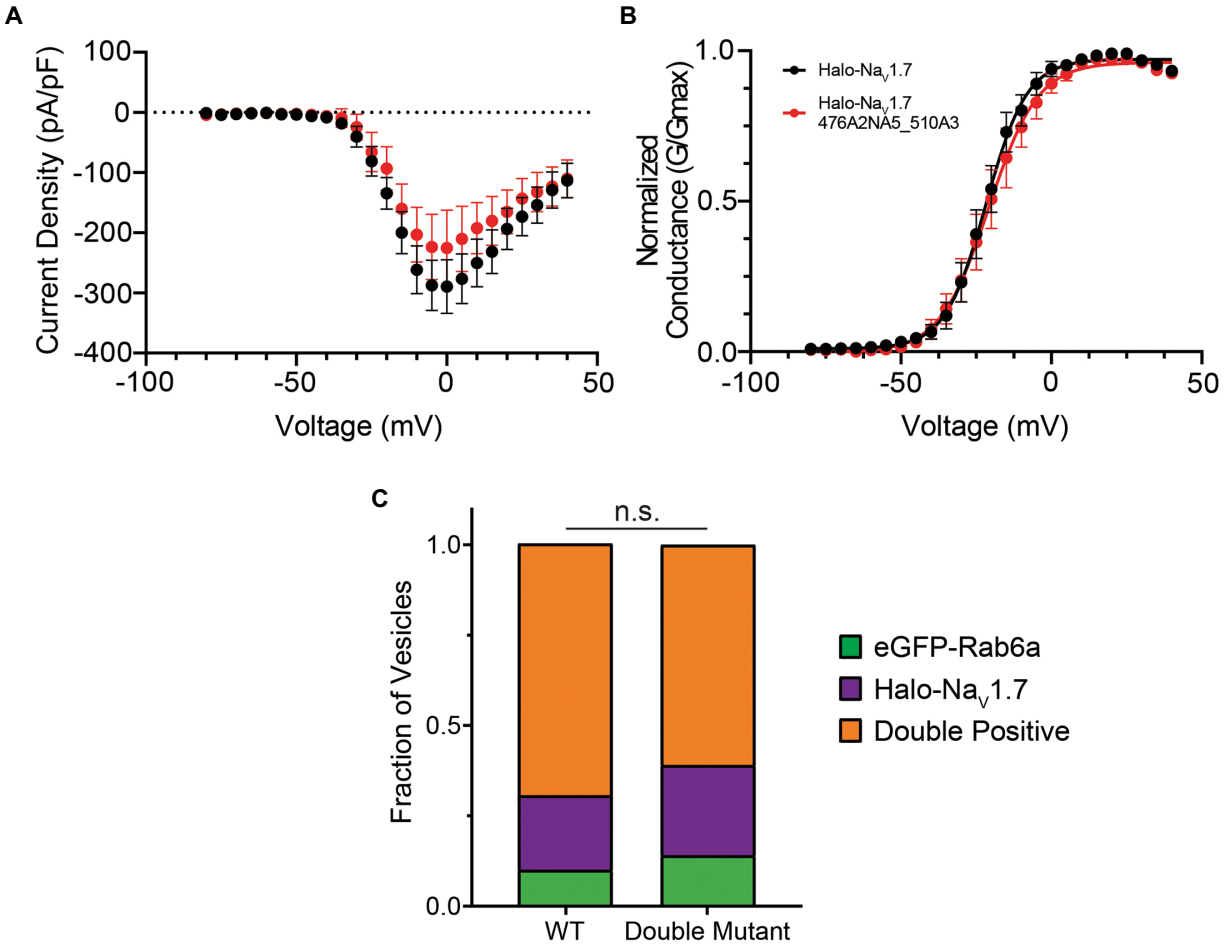
Mutating both polybasic motifs in the L1 of Na_V_1.7 does not affect channel gating or co-trafficking with Rab6a. **(A)** The current–voltage relationship of Halo-Na_V_1.7 476A2NA5_510A3 is similar to that of WT Halo-Na_V_1.7 as assessed by automated voltage-clamp. Peak current density was not significantly different between the two channels (−225.4 ± 63.2 vs. −289.3 ± 44.5 pA/pF for Halo-Na_V_1.7 476A2NA5_510A3 (*n* = 19) and WT Halo-Na_V_1.7 (*n* = 26), respectively; p = 0.40 by unpaired *t*-test). **(B)** The voltage-dependence of activation is not significantly different between the two channels. Conductance-voltage relationships were fit by Equation 3 (Supplementary material). Half-maximal activation voltages were similar between the two constructs (−21.7 ± 0.5 mV vs. −20.8 ± 0.7 mV, for WT Halo-Na_V_1.7 (*n* = 26) and Halo-Na_V_1.7 476A2NA5_510A3 (*n* = 19) respectively; *p* = 0.29 by unpaired *t*-test). **(C)** The fraction of vesicles that demonstrate co-transport of Na_V_1.7 and Rab6a is not affected by mutation of both polybasic motifs. Two-color time lapse imaging was performed. Kymographs were created for each construct-Rab6a pair, and vesicle tracks were visually inspected and classified as Rab only, Na_V_1.7 only, or both (*p* = 0.47; *n* = 125–145 vesicles per channel, 3 cultures, 7 axons per culture). ”ns” stands for non-significant (*p*>0.05).

To probe this hypothesis, we also generated a double mutant construct where both the 476RRNRRKKK and 510RRK motifs were substituted by alanine residues (Na_V_1.7 476A2NA5_510A3). We first verified that the double-mutant channel displayed electrophysiologic parameters similar to WT (Figures 3A,B; Supplementary Figure S1B). We then repeated our experiments using the OPAL imaging technique. Similar to our results with the single mutants, mutation of both polybasic motifs did not result in a statistically significant difference in vesicle composition (*p* = 0.47 by χ^2^ test). In DRG neurons expressing the double mutant channel, 61% of vesicles were doubly positive for Halo-Na_V_1.7 and eGFP-Rab6a, 25% positive for the Halo-tagged channel alone, and 14% positive for eGFP-Rab6a alone (Figure 3C).

### 3.3. Polybasic motifs 476RRNRRKKK and 510RRK do not affect velocity or flux of anterograde trafficking of Na_V_1.7

We next tested if mutations in the L1 polybasic motifs resulted in fewer vesicles reaching the axonal chamber (as this could indicate retention in the soma). There were no significant differences in vesicle flux between the three channel constructs (median [IQR] 12.5 (Dib-Hajj et al., 2010) vs. 10.5 (Basbaum et al., 2009) vs. 10 (Dib-Hajj et al., 2010) vesicles/min/axon for Halo-Na_V_1.7, Halo-Na_V_1.7 476A2NA5, and Halo-Na_V_1.7 510A3, respectively; *p* = 0.31 by Kruskal-Wallis ANOVA; Figure 2D).

Since Rab6a is an interacting partner with kinesin motor proteins (Echard et al., 1998; Lee et al., 2015), we also measured vesicle velocity. There were no statistically significant differences in vesicle velocity between the three channel constructs (median [IQR] 0.87 [1.58] vs. 1.25 [1.76] vs. 0.94 [1.69] mm/s for Halo-Na_V_1.7, Halo-Na_V_1.7 476A2NA5, and Halo-Na_V_1.7 510A3, respectively; *p* = 0.52 by Kruskal-Wallis ANOVA; Figure 2E).

### 3.4. Polybasic motifs 476RRNRRKKK and 510RRK do not affect accumulation of Na_V_1.7 at the distal axonal terminal

**Figure 4:**
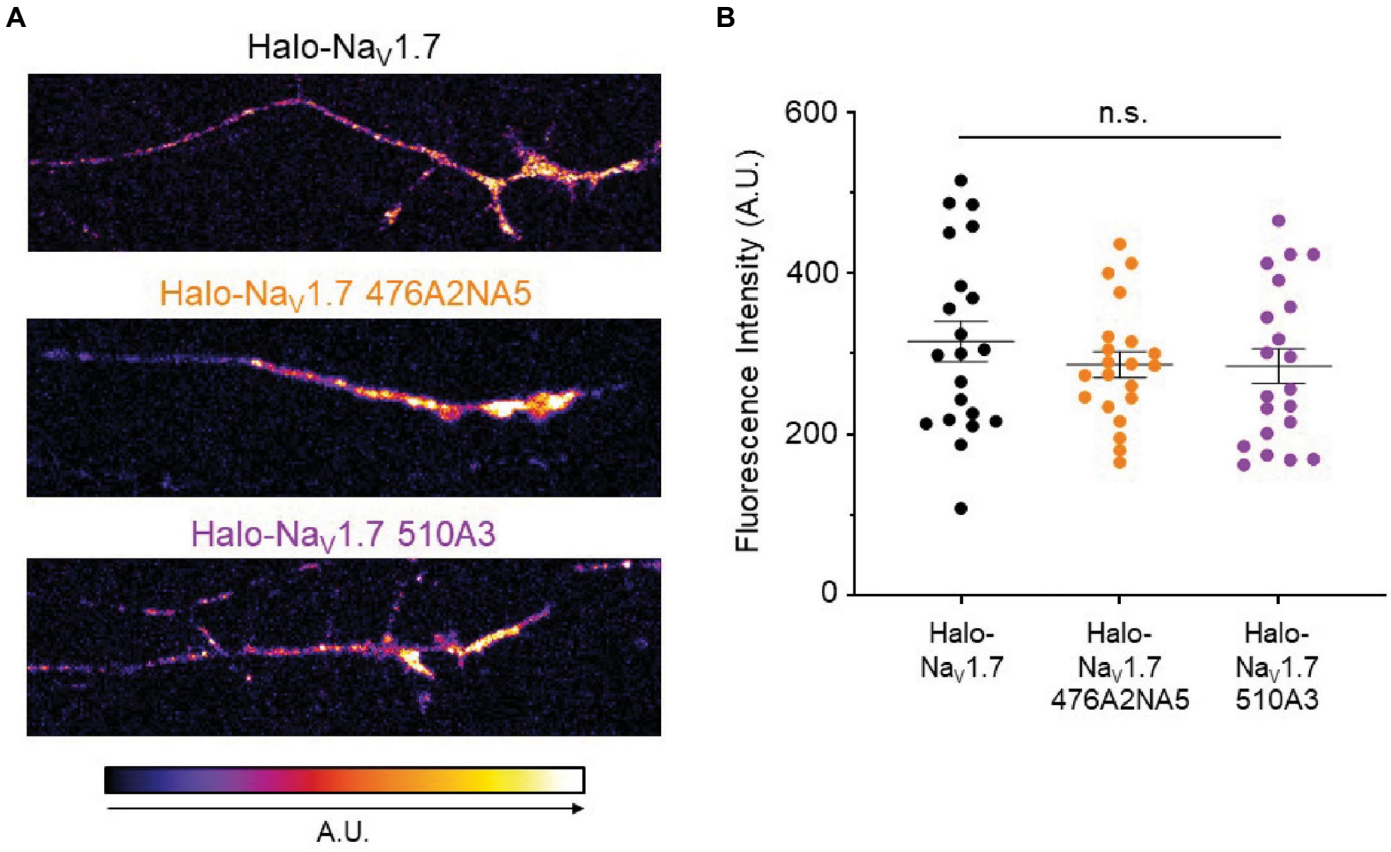
Mutations of the polybasic motifs in the Na_V_1.7 L1 do not affect accumulation of the channel at the axonal terminal. **(A)** DRG neurons transfected with either Halo-Na_V_1.7, Halo-Na_V_1.7 476A2NA5, or Halo-Na_V_1.7 510A3 were labeled with a cell-impermeable Halo dye after 6 days in culture. Fluorescent signal was quantified in ImageJ and mapped to the look-up table shown at the bottom of the left panel **(B).** There was no significant difference in fluorescent signal from Na_V_1.7 at the distal axonal surface between DRG neurons transfected with either Halo-Na_V_1.7 (*n* = 21, from 3 cultures), Halo-Na_V_1.7 476A2NA5 (*n* = 21, from 3 cultures), or Halo-Na_V_1.7 510A3 (*n* = 21, from 3 cultures). Bars indicate mean ± SEM. ”ns” stands for non-significant (*p*>0.05).

Since Na_V_1.7 density at the axonal terminal increases in some settings of neuronal hyperexcitability (Akin et al., 2019, 2021), we sought to investigate whether mutation of the L1 polybasic motifs resulted in a difference in Na_V_1.7 accumulation at the surface of the distal axon. We transfected DRG neurons with either Halo-Na_V_1.7, Halo-Na_V_1.7 476A2NA5, or Halo-Na_V_1.7 510A3. After 6 days *in vitro*, we used a cell-impermeable Halo dye (JF-635i) to label existing Na_V_1.7 channels at the axonal surface (Figure 4A). We then used spinning-disk confocal microscopy to image the axonal terminals and quantified fluorescent signals coming from labeled Na_V_1.7 channels. There was no significant difference between Na_V_1.7 fluorescence intensity in DRG neurons transfected with the three constructs (315.1 ± 25.01, 286.4 ± 16.05, 284.6 ± 21.4 A.U., for Halo-Na_V_1.7, Halo-Na_V_1.7 476A2NA5, and Halo-Na_V_1.7 510A3, respectively; *p* = 0.61 by Kruskal-Wallis ANOVA; Figure 4B).

## 4. Discussion

Na_V_1.7 sodium channels play a major role in regulating the threshold of pain-signaling peripheral neurons (Dib-Hajj et al., 2013; Bennett et al., 2019; Dib-Hajj and Waxman, 2019). Identifying the basis for the selective sorting of Na_V_1.7 into Rab6a positive vesicles is of interest because this interaction could be harnessed for the reduction of anterograde Na_V_1.7 trafficking to the neuronal surface. Indeed, disruption of the Rab-channel interaction has pharmaceutical precedence, as the anticonvulsants Gabapentin and Pregabalin inhibit Rab-dependent recycling of voltage-gated calcium channel subunits and subsequently reduce calcium currents, decreasing painful neurotransmission (Tran-Van-Minh and Dolphin, 2010; Patel and Dickenson, 2016).

The principal question answered by this investigation is whether two polybasic motifs in the L1 of human-Na_V_1.7 (476RRNRRKKK and 510RRK) are important in Rab6a-mediated channel trafficking in DRG neurons. Surprisingly, mutation of either or both polybasic domains did not affect co-trafficking of the channel with Rab6a. The polybasic motif mutants exhibited similar proportions of vesicles positive for both Rab6a and Na_V_1.7, as well as the individual proteins. Additionally, the polybasic motifs do not appear to be critical for Na_V_1.7 trafficking to the plasma membrane in general. At the soma, whole-cell Na_V_1.7 currents were not significantly different between the mutants and the WT channel. Though our electrophysiologic studies show that somatic-trafficking of Na_V_1.7 remains intact following PBM mutation, delivery of membrane-proteins to different neuronal compartments is polarized (Lasiecka and Winckler, 2011). Our imaging studies complement our electrophysiologic findings by showing that, at the axonal terminal, similar amounts of Na_V_1.7 are visualized in DRG neurons expressing either mutant or WT channels.

Cytoplasmic domains of membrane proteins frequently contain basic residues that direct a variety of vesicular fates including retention, sorting, and export (Jackson et al., 1990; Andersson et al., 1999; Michelsen et al., 2005). Pertaining specifically to vesicle export, Na_V_1.7 has been shown to preferentially travel in vesicles that contain the Rab6a GTPase (Akin et al., 2019). Polybasic motifs have been shown to be necessary for Rab-mediated Golgi-export and membrane delivery in other plasma membrane proteins (Parent et al., 2009; Parmar et al., 2014a,b; Parmar and Duncan, 2016). In the case of the p14 FAST protein, the polybasic motifs acted as electrostatic interaction motifs that conferred a Golgi-export phenotype to a protein that would otherwise be retained in the Golgi. Basic residues in an ion channel have also previously been linked to Golgi export – the inward rectifier potassium channel Kir2.1 contains two N-terminal basic residues that, when mutated, result in significantly decreased channel delivery to the plasma membrane (Stockklausner and Klöcker, 2003).

Rab GTPase interactions with cargo proteins have been shown to be a feature of Rab-regulated intracellular trafficking (Aloisi and Bucci, 2013). However, no consensus motif has been discovered for Rab-protein interactions. Polybasic motifs were an attractive candidate due to their presence in other cargo protein-Rab interactions. In the β2 adrenergic receptor, for example, basic residues in the C-terminal tail are critical for the interaction with the Rab protein partner (Parent et al., 2009). An MLERK motif in the cytoplasmic domains of the TRPV5 and TRPV6 ion channels is required for the interaction of those channels with Rab11 as well (van de Graaf et al., 2006). However, based on our data, this does not appear to be the case for an interaction between Rab6a and the two PBMs in L1 of Na_V_1.7. There are several potential explanations for this. For one, it is possible that Rab6a and Na_V_1.7 interact through one or more adaptor proteins, and that the polybasic motifs in the Na_V_1.7 L1 are not required for any of these interactions. Alternatively, even though Rab6a frequently co-localizes with Na_V_1.7, these two proteins may not be mechanistically linked. There have been no studies demonstrating a direct interaction between Rab6a and Na_V_1.7, and anterogradely moving vesicles carrying Na_V_1.7 have been shown to contain a variety of other proteins (Higerd-Rusli et al., 2022). If Rab6a and Na_V_1.7 do indeed interact, more work would need to be done to tease out the mechanism of this interaction as it would be of potential therapeutic interest. Finally, it is possible that other Rab GTPases may co-localize with Na_V_1.7 in anterograde vesicles. While to-date, only Rab6a has been confirmed to co-transport with Na_V_1.7 in anterograde vesicles, there are dozens of other Rab isoforms that have not been tested (Usoskin et al., 2015; Akin et al., 2019). Still, the polybasic motifs may not be physiologically relevant to these hypothetical associations since expression and function of the channel are preserved in the absence of the PBMs.

Polybasic motifs are also required for plasma membrane localization in several proteins (Aguilar et al., 2007; Crouthamel et al., 2008; Barbosa et al., 2016). That is, PBMs are required for insertion into the membrane directly. It is postulated that polybasic clusters interact electrostatically with negatively charged lipids on the plasma membrane (Heo et al., 2006; Li et al., 2014). A polybasic motif (RRR) in the L1 of Na_V_1.8 has been shown to act as an endoplasmic reticulum (ER) retention motif, which is masked by interaction with the cytoplasmic tail of the β3 sodium channel subunit (Zhang et al., 2008). The study also showed that neutralizing mutations in this motif increased the current density of the channel and eliminated the sparing effect of the β3 subunit.

The polybasic motifs at AAs 476 and 510 are conserved in Na_V_1.7 across many species, thus it is a peculiar finding that complete ablation of these residues has seemingly no effect on channel function. In our experiments, Halo-Na_V_1.7 mutant channels were still detected in similar number to WT channels on axon terminals (Figure 4). Though we did not specifically interrogate dynamics of channel insertion in this study, our results indicate that the L1 PBMs are not necessary for Na_V_1.7 to be delivered to the neuronal plasma membrane. It is possible that these motifs are important for interactions with other protein partners of Na_V_1.7, but that these effects only become clear in certain cellular situations (e.g., inflammation).

Our mutant constructs were devoid of several charged residues in a region of the channel that has previously been linked to control of gating properties (Dib-Hajj et al., 2013; Ahern et al., 2015).The L1 of Na_V_1.7 contains four sites at which MAPK1 and MAPK3 phosphorylate the channel. This phosphorylation produces a hyperpolarizing shift in channel activation (Stamboulian et al., 2010). Even with our mutants containing such large changes in sequence, the channels retained WT gating properties and current density (Figures 2, 4A,B).

Our methodology provides a powerful way to investigate trafficking of single ion channels in real time. However, there are several limitations to our approach. For one, the HaloTag system does not allow for accurate resolution of events in the neuronal soma because of the large cytoplasmic pool of channels in cytoplasmic organelles, preventing the ability to detect vesicles moving between these compartments. Therefore, we were only able to investigate the effects of the L1 polybasic motifs on axonal trafficking and delivery. Second, we make use of an overexpression system in our study. It is possible that overexpression of these proteins results in non-physiologic co-trafficking and surface expression patterns that are masking the true effects of PBM ablation. To solve this problem, we could make use of a model system that endogenously expresses Halo-tagged Na_V_1.7, such as a cell line modified with CRISPR gene-editing to express the Halo-tag on endogenously produced Na_v_1.7 channels (Caine et al., 2020). Additionally, we have demonstrated that overexpression does not cause proteins to be trafficked indiscriminately (Akin et al., 2019; Higerd-Rusli et al., 2022). We must also note that measuring current density by voltage-clamp recording in this study was performed in a heterologous expression system, which does not always reflect how ion channels behave in a neuronal context. However, when coupled with our findings by imaging in DRG neurons, we do not have reason to suspect that there would be differences in current density if the electrophysiology was conducted in DRG neurons instead of HEK293 cells.

Exploration of Na_V_1.7-linked therapeutics that do not rely on blocking channel conductance as a mechanism for analgesia is an emerging area of research that could yield novel strategies and compounds for the alleviation of pain (Shantanu et al., 2019; Wulff et al., 2019; Sun et al., 2022). The principal contributions of our study are that 1) polybasic motifs in the L1 of human Na_V_1.7 do not contribute to channel trafficking in DRG neurons, 2) Rab6a co-trafficking with Na_V_1.7 persists in the absence of the PBMs in L1, and 3) the conserved polybasic clusters in Na_V_1.7 are not critical for channel insertion or function in the plasma membrane. Future research should continue to interrogate potential sites of channel-partner interaction (including interactions with Rab6a and other Rab GTPases).

## Data availability statement

The raw data supporting the conclusions of this article will be made available by the authors, without undue reservation.

## Ethics statement

The animal study was reviewed and approved by Institutional Animal Care and Use Committee at the Department of Veterans Affairs West Haven Medical Center.

## Author contributions

ST designed research, performed research, analyzed data, and wrote the manuscript. NS performed research. GH-R performed research and critically reviewed the manuscript. SL and FD-H provided critical research reagents. SW and SD-H designed research, wrote the manuscript, and critically reviewed and edited the manuscript. All authors contributed to the article and approved the submitted version.

## Funding

This work was supported by Merit Review Awards B9253-C and BX004899 from the U.S. Dept. of Veterans Affairs Rehabilitation Research and Development Service and Biomedical Laboratory Research and Development Service, respectively (SW and SD-H). The Center for Neuroscience and Regeneration Research is a Collaboration of the Paralyzed Veterans of America with Yale University. ST and GH-R were supported by NIH/NIGMS Medical Scientist Training Program T32GM007205. GH-R was supported by NIH/NINDS 1F31NS122417–01.

## Conflict of interest

The authors declare that the research was conducted in the absence of any commercial or financial relationships that could be construed as a potential conflict of interest.

## Publisher’s note

All claims expressed in this article are solely those of the authors and do not necessarily represent those of their affiliated organizations, or those of the publisher, the editors and the reviewers. Any product that may be evaluated in this article, or claim that may be made by its manufacturer, is not guaranteed or endorsed by the publisher.

## Footnotes

^1^ https://github.com/ycnrr
