# Supplementary material for: Conserved but not critical: Trafficking and function of NaV1.7 are independent of highly conserved polybasic motifs
Source: Front Mol Neurosci. 2023 Mar 16;16:1161028. doi: 10.3389/fnmol.2023.1161028 (PMC10060856; doi:10.3389/fnmol.2023.1161028)
Supplement: Supplementary file 1 [file Data_Sheet_1.docx]

**Supplementary Materials

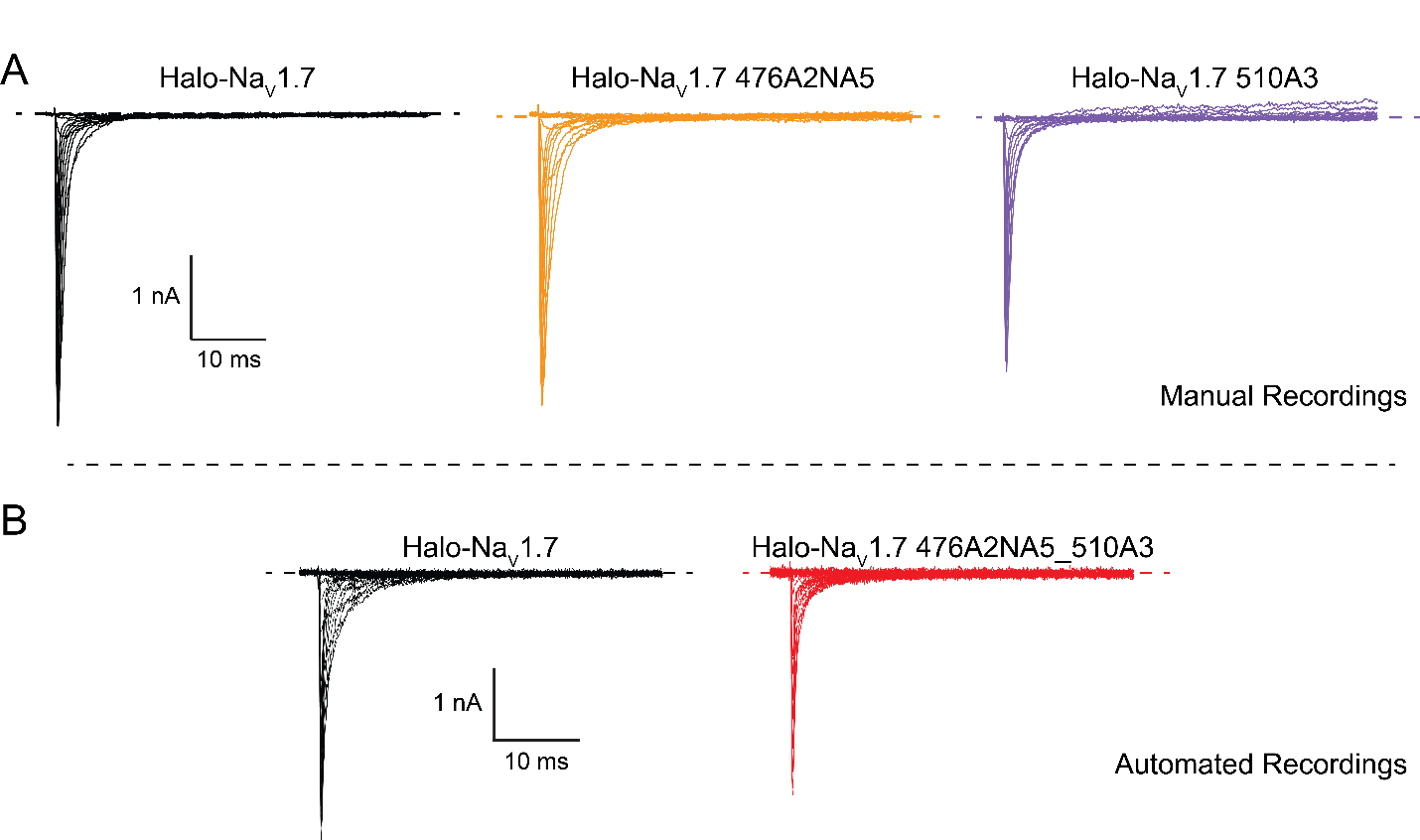
**

**Figure S1. Representative electrophysiological recordings of Halo-tagged Na_V_1.7 constructs.** (A) Representative currents from manual voltage-clamp of HEK293 cells transfected with Halo-Na_V_1.7(left), Halo-Na_V_1.7 476A2NA5 (middle), or Halo-Na_V_1.7 510A3. (B) Representative

currents from automated voltage-clamp of HEK293 cells transfected with Halo-Na_V_1.7(left) or Halo-Na_V_1.7 476A2NA5_510A3 (right).

**Supplementary Methods**

**DRG Cultures:**
Neonatal rats were selected randomly and thus included males and females. Dissected DRGs were incubated at 37°C for 20 min in complete saline solution (CSS) [in mM: 137 NaCl, 5.3 KCl, 1 MgCl2, 25 sorbitol, 3 CaCl_2_, and 10 HEPES (pH 7.2), adjusted with NaOH], supplemented with 0.6 EDTA and collagenase A (1.5 mg/ml). Following this, DRGs were then incubated for 20 min at 37°C in CSS containing collagenase D (1.5 mg/mL;), 0.6 EDTA, and papain (30 U/ml). DRGs were centrifuged and triturated in 1 ml of DRG culture medium DMEM/F12 (1:1) with penicillin (100 U/ml), streptomycin (0.1 mg/ml), 2 L-glutamine, and 10% FBS containing BSA (1.5 mg/ml; low endotoxin) and trypsin inhibitor (1.5 mg/ml). The suspended neurons were then filtered through a 70 μm nylon mesh cell strainer to remove debris, and then washed with DRG culture medium. For transfections, DRG neurons were pelleted and gently resuspended with 20 μl of Nucleofector solution. Then, the cell suspension was mixed with the appropriate plasmids. Neurons were transfected using Nucleofector IIS Electroporator. After electroporation, 100 μl of calcium-free DMEM was added, and cells were incubated at 37°C for 5 min in a 95% air/5% CO2 (v/v) incubator to allow neurons to recover. The cell mixture was then diluted with DRG medium containing BSA (1.5 mg/ml) and trypsin inhibitor (1.5 mg/ml). Rat DRGs were seeded onto 35 mm glass-bottom dishes commercially coated with poly-D-lysine and with laminin (10 mg/ml for 2 h at 37°C), or onto the somatic chamber of microfluidic chambers (MFCs). DRGs cultured on coverslips were incubated at 37°C for 45 min to allow DRG neurons to attach. Then, DRG medium was added to each well to a final volume of 1.5 ml (in 35 mm glass-bottom dishes), or ∼350 μl (in the somatic chamber of MFCs) individually. DRG neurons were maintained at 37°C in a 95% air/5% CO2 (v/v) incubator before use.

**Electrophysiology**

Borosilicate pipettes (1.0-2.0 MΩ) were filled with an internal solution containing (in mM): 140 CsF, 10 NaCl, 1.1 EGTA, 10 HEPES, 20 Dextrose (pH 7.3 with CsOH, adjusted to 310 mOsm/L with dextrose). External bath solution contained (in mM): 140 NaCl, 20 TEA-Cl, 3 KCl, 1 CaCl_2_, 1 MgCl_2_, 20 HEPES, 20 Dextrose (pH 7.3 with NaOH, adjusted to 320 mOsm/L with dextrose). Voltage clamp protocols were initiated 5 minutes after establishing whole-cell configuration on an EPC-10 USB amplifier (HEKA Instruments) and acquired using PatchMaster Next software. Electronic compensation was used to reduce the effective series resistance. Linear components of leak and capacitive currents were corrected with -P/6 online subtraction protocols. Filtering was at 2.9 kHz and acquisition was at 50 kHz. Cell capacitance was determined by integration of a transient from -120 mV to -110 mV using the PatchMaster Next software. Voltage errors were minimized with 60-90% series resistance compensation, and only cells with <3 mV voltage error after compensation were included for analysis.

To examine the current-voltage (I-V) relationship, a series of step depolarizations from -80 to +40 mV in 5-mV increments were applied from a holding potential of -120 mV at 10-s interpulse intervals.

To examine the inactivation properties of the channel constructs, conditioning pulses of 500 ms duration ranging from -140 to +30 mV in 10 mV increments were applied from a holding potential of -120 mV. Following the conditioning pulse, a 40 ms test pulse to -10 mV was applied to determine the fraction of sodium channels still available for opening. Peak inward currents were recorded and fit by Supplementary Equation 3.

*Automated Voltage Clamp Experiments*

The gating properties of the Halo-Na_V_1.7 476A2NA5_510A3 was evaluated by automated voltage-clamp of transiently transfected HEK cells. Plasmids encoding Halo-Na_V_1.7 476AANAAAAA_510AAA or WT Halo-Na_V_1.7 (as the control condition) were introduced into HEK 293 cells via lipid-based transfection (Lipofectamine 3000, ThermoFisher). Cells were harvested into a cell suspension 48 hours post-transfection for recordings by an automated electrophysiology robot (Qube 384, Sophion Biosciences). A detailed protocol for this type of high-throughput protocol is described elsewhere ^40–42^.

The solutions used in Qube experiments are as recommended by Sophion. The intracellular solution contained the following (in mM): 140 CsF, 1 EGTA, 10 HEPES, 10 NaCl (pH 7.3 with CsOH, adjusted to 310 mOsm/L with dextrose). The extracellular solution contained the following (in mM): 145 NaCl, 4 KCl, 2 CaCl_2_, 1 MgCl_2_, 10 HEPES (pH 7.4 with NaOH, adjusted to 305 mOsm/L with dextrose).

Voltage-clamp protocols were designed to mimic those used for manual patch clamp. Like manual recordings, activation protocols were run before inactivation protocols to prevent time-dependent bias. Series resistance compensation was set to 90%. A cell was deemed positive for Na_V_ current expression if the peak inward current during the activation protocol exceeded -300 pA. Additional inclusion criteria included a reversal potential between 10 and 80 mV, and a Boltzmann slope factor >4 mV ^18^. Peak inward currents were fit by supplementary equation 1 as described above. Activation curves were generating by applying supplementary equations 2 and 3 as described above. Inactivation curves were generated by applying supplementary equation 3 above.

**Electrophysiology Curve Fitting**

Peak and normalized I-V curves were fitted according to:

$I= \frac{G_{max}(V-V_{rev})}{(1+e^{\frac{V_{0.5}-V}{k_{G}}})}$ (Supplementary Equation 1)

where I is the peak or normalized current for the test potential V, V_rev_ is the reversal potential, V_0.5_ is the voltage of half-maximal activation, and k_G_ is the Boltzmann slope factor.

Inward currents were transformed to conductance using the equation:

$G= \frac{I}{(V_{m}-E_{Na})}$ (Supplementary Equation 2)

Where I is the inward current, V_m_ is the test potential, and E_Na_ is the reversal potential for Na^+^.

The conductance at each voltage was normalized to the maximum conductance and fit with the following Boltzmann equation to derive the activation curve.

$G=G_{min}+\frac{(G_{max}-G_{min})}{1+e^{\frac{(V_{m}-V_{0.5})}{k}}}$ (Supplementary Equation 3).

Where G_max_ is the maximal conductance, G_min_ is the minimal conductance, V_m_ is the test potential, V_0.5_ is the voltage of half-maximal activation, and k is the Boltzmann slope factor.

Activation kinetics were fit with a single exponential function:

$I=I_{0}[e^{\left( \frac{-t}{\tau_{act}} \right)}]$ (Supplementary Equation 4).

Where I_0_ is the initial inward current, t is the time elapsed, and τ_act_ is the time constant of activation.
